# Newly discovered *Staphylococcus aureus* serine hydrolase probe and drug targets

**DOI:** 10.5599/admet.1137

**Published:** 2021-10-28

**Authors:** Matthias Fellner

**Affiliations:** Department of Biochemistry, School of Biomedical Sciences, University of Otago, Dunedin, New Zealand. matthias.fellner@otago.ac.nz; Tel.: +64 34797897

**Keywords:** Inhibitor, biofilm, fluorophosphonate

## Abstract

There is an urgent need for new diagnosis and treatment options for the bacterial pathogen Staphylococcus aureus. This review will summarize data on ten recently discovered biofilm-associated serine hydrolases called fluorophosphonate-binding hydrolases (FphA-J). Based on the summarized findings, many of these proteins represent intriguing new targets for probe and drug development.

## Introduction

Antimicrobial resistance presents a major challenge to public health, with the past year accelerating the problem [1, 2]. This is especially true for infections by the bacterial pathogen *Staphylococcus aureus*, which are a major cause of mortality, often linked to community-acquired drug-resistant strains (MRSA) [3]. This calls for an urgent need to find new solutions for effective diagnosis and treatment options to overcome resistance to avoid depletion of our arsenal of antibiotics. New protein targets within *S. aureus* are required for the development of efficient diagnostic probes both for imaging applications and as a treatment strategy to block productive infection by the bacteria without pressuring the organism to select resistance mutants. A fluorophosphonate-based activity-based probe identified ten previously uncharacterized active serine hydrolases in *S. aureus* during biofilm-promoting growth conditions, which could fill this need. These were named fluorophoshonate-binding hydrolases (Fphs), with sequential letters for each enzyme based on their predicted size (52 kD FphA – 22 kD FphJ) [4]. All are members of the α/β hydrolase superfamily, characterized by a core of eight β-strands connected by several α helices with an active site triad of serine-histidine- aspartate or glutamate. The nucleophilic serine is used for the hydrolysis of substrates and can easily and specifically be targeted by small molecules [5, 6]. In general, these proteins play important roles in processing of metabolites, peptides and lipids as a means of controlling cell signalling and metabolism; however, to date the biological function remains unknown for all Fphs and only the structure of FphF has been determined [6, 7]. Their active state during biofilm-forming conditions makes them accessible to modification by chemical inhibitors that can be developed into probes and drugs. Such new compounds have the potential for diagnosis, monitoring or treatment of *S. aureus* infections. This review will summarize our current knowledge about each fph gene and Fph protein with the gene name of the reference *S. aureus* strain NCTC 8325, also listed in the headline.

## Fph Proteins

### FphA - SAOUHSC_02751

**Figure 1.**
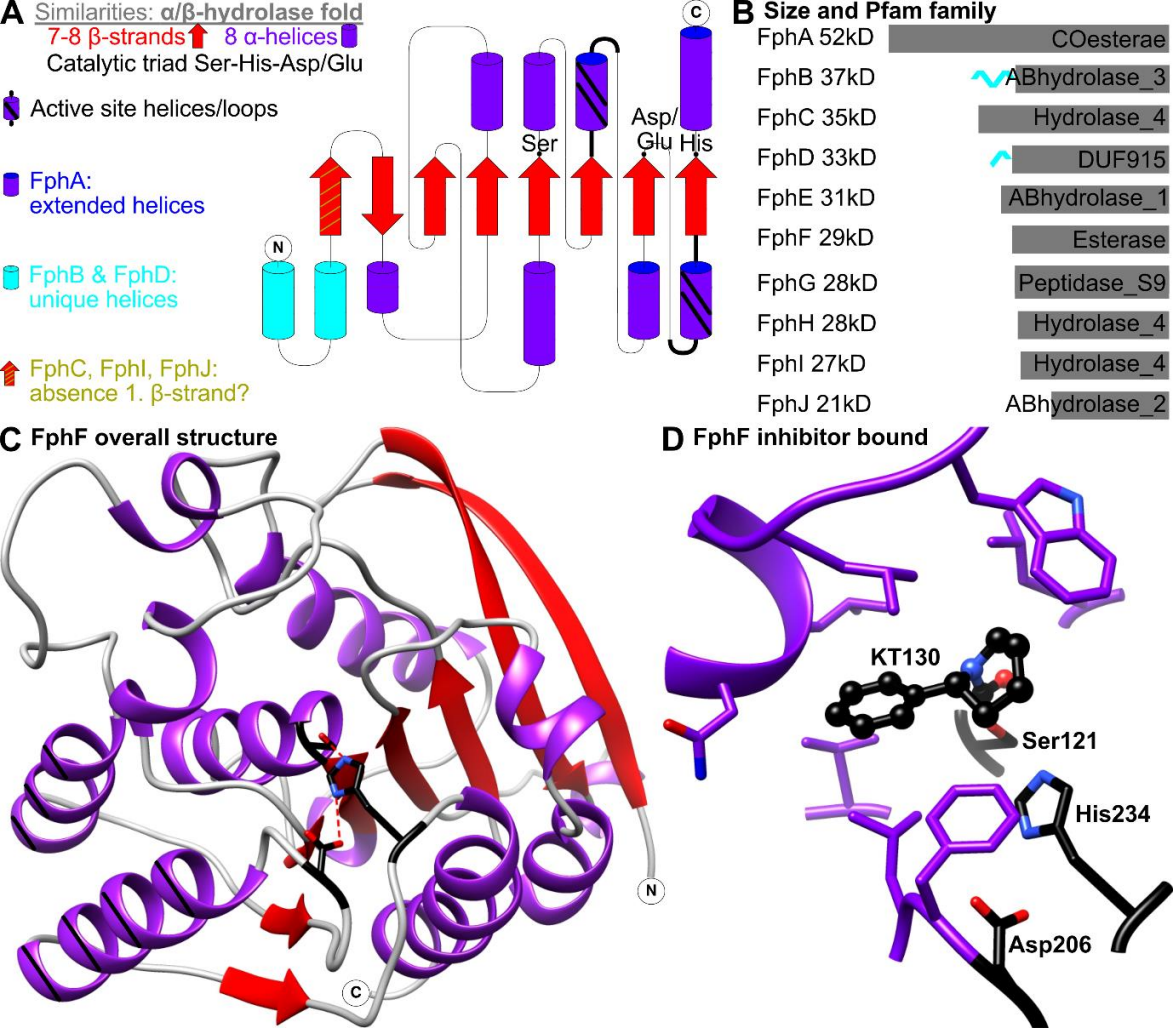
(a) Overview of the α/β hydrolase fold shared by all Fph proteins. Shared and unique features color coding illustrated. **(b)** Molecular size and hydrolase domain Pfam family association for FphA-J. Grey box represents the predicted size of the hydrolase domain with unique helices in cyan. **(c)** FphF structure as an example of the hydrolase domain (PDB ID 6VH9). Active site triad residues in black with hydrogen bonds as red dotted lines. α helices in purple, active site helices marked black. Core β-strands in red. **(d)** FphF KT130 inhibitor bound to the hydrophobic active site pocket. Residues of this pocked in purple, with active site triad and covalent bound KT130 in black.

FphA is significantly larger than the other Fph proteins at 52kD (Fig. 1A; secondary structure figure made with TopDraw [8]). The size increase stems from a possible additional β-strand and especially from large extensions of the connecting loops and helices to the core β sheet. FphA is predicted to be a carboxylesterase (Fig. 1B, Pfam [9]) and structure prediction suggests a narrow substrate profile due to the range of hydrophobic and hydrophilic residues in a small but surface-exposed active site pocket [6]. A library screen of 1,2,3-triazole urea molecules failed to identify a highly specific inhibitor for FphA with FphA showing a broad range of low level of inhibition compared to a more defined profile of specific hits for other investigated Fph proteins [10].

### FphB - SAOUHSC_02844

FphB stands out with predicted additional N-terminal extensions that contain three large α-helices preceding the core hydrolase fold (Fig. 1A and 1B). Function and structure predictions do not give a confident suggestion of the role of these helices, with possible roles being membrane association or active-site capping [4, 6]. FphB may have a role in stress responses as transcriptomic datasets showed a moderate upregulation of fphB gene transcription (~1.8–2.9-fold) upon exposure to various cell-wall-acting antibiotics and antimicrobial peptides (locus tags SA2323 [11-13] and SA2549 [14], respectively); to neutrophil-associated azurophilic granules, hydrogen peroxide, hypochlorus acid (locus tag MW2456) [15]; to acid shock (locus tag SA2549) [16]; and antibacterial skin fatty acids (temporary upregulation, locus tag SA2323) [17]. Another study showed FphB upregulation comparing *S. aureus* exponentially and stationary phases in media, in a S9 human bronchial epithelial cell line infection model and a murine pneumonia model [18].

Using commercially available fluorogenic substrates revealed that FphB has cleavage-specificity towards esterase substrates with a preference for C4 > C7 > C8 fatty acid esters and failing to cleave C2 or C10 and longer substrates. For FphB, small molecule inhibitor screening identified a chloroisocoumarin-based covalent inhibitor JCP251 [4] and a triazole-based inhibitor AA395 [10], both with specificity over the other Fph proteins. JCP251 effectively reduced infectivity in a mouse model, suggesting that it may be a viable therapeutic target for the treatment or management of Staphylococcus infections. This inhibitor also had a fast inactivation rate when the esterase activity was tested *in vitro*. JCP251 was subsequential modified into a fluorescent probe JCP251-bT, which only reduced the inactivation rate 3-fold. In live *S. aureus* cells, this probe only labelled FphB; it also did not label any targets in several other bacterial pathogens and predominantly labelled FphB in live bacteria cocultured with peripheral mononuclear cells and mouse RAW264.7 macrophage cells. This allows for visualization of probe-labelled bacteria over weak background fluorescence caused by nonspecific probe uptake and/or weak labelling of host cell proteins. Various bacteria-host cell coculture experiments suggested that *S. aureus* (strains ATCC35556 and Newman) regulates FphB activity in response to host-cell-derived signals and that FphB is not secreted into the culture medium. Compared with a cytosolic GFP signal, FphB labelling is concentrated in specific regions of the cell envelope, especially in the septal cross-wall, suggesting that FphB might act on substrates located in the bacterial cell wall. However, the FphB-deficient strain had no growth defect in liquid culture and showed a similar doubling time to the wild-type, ruling out a general role in cell division. However, a ~10- to 100-fold decrease in bacterial loads in liver and heart tissues of infected mice from wild-type to the FphB-deficient strain suggests a critical role in the colonization of these specific organs as no phenotype was observed in kidneys. Pre-treatment of wild type with JCP251 showed a similar reduction of bacterial burden in the liver to levels comparable to those observed upon infection with the FphB-deficient strain. Overall, FphB is an important virulence factor in the early stages of infections in the heart and liver but not the kidneys, potentially due to different environments during initial colonization. The inhibitory effect of JCP251 demonstrates that FphB is an ideal target for the development of small molecules that could stop the spread of primary infection to other sites that cause increased morbidity and mortality [4].

Studies on the 58 % sequence identical FphB homolog from the commensal *S. epidermidis* showed similar substrate preferences, with an additional ability to process an acetate ester, with inhibition of the protein also not influencing the growth of the bacteria. However, in contrast to FphB in *S. aureus*, the homolog in *S. epidermidis* appears to not have a role in colonization *in vivo* based on preliminary studies in skin-infected mice [19].

### FphC - SAOUHSC_01279

FphC and FphG were most difficult to label among the Fph proteins with the biotinylated and fluorescent probes used to initially characterize the Fph proteins, possibly due to comparable lower abundance and beyond their initial discovery, not much is known about these two hydrolases [4]. Interestingly FphC, FphG and FphH are the most conserved Fph proteins in the genus Staphylococcus [19]. Structure prediction suggests that FphC may lack the first core β-strand (Fig. 1A) but otherwise shows a typical α/β hydrolase fold with a helix capping the active site [6]. FphC was upregulated comparing *S. aureus* exponentially and stationary phases in media [18].

### FphD - SAOUHSC_01279

FphD is the only Fph protein with a significant structural homology hit in the protein data bank, with the 63 % FphD homolog from *S. epidermidis* (PDB ID 3FLE, unpublished). This structure shows a large surface exposed active site with mostly hydrophobic residues, however, the N-terminal is missing in the structure, and FphD is predicted to contain several helices here (Fig. 1A and 1B). Similar to FphB these helices could serve various functions in FphD, however, capping the active site is more likely here due to the large surface exposed area [6]. Beyond this, not much is known about FphD, its expression may be linked to FphH based on gene knockout studies [4] and FphD was downregulated in an S9 bronchial epithelial cell infection model [18].

### FphE - SAOUHSC_02900

Analysis of 50 different species in the genus Staphylococcus showed that FphE is the least conserved Fph protein, with less than half containing a sequence relevant homolog [19]. Studies suggest that FphE could be an *S. aureus* specific driver of virulence, with a fphE transposon mutant showing a small, though statistically significant, reduction of bacterial loads in the mice livers compared to wild type, with no reduction in the hearts or kidneys [20]. FphE and FphG were the only Fph proteins identified to bind β-lactam probes [20, 21]. A screening of 1,2,3-triazole urea compounds identified inhibitors for FphE, one was modified into a fluorescent probe, resulting in a highly specific cellular probe to study FphE activity or to sort bacterial populations based on probe-labelling status [10]. In both *S. aureus* USA300 and Newman strains, FphE-specific labelling was highest in cells growing under biofilm-forming conditions compared to exponential growth and in the late stationary phase. Throughout all culture stages, FphE labelling was higher in USA300 compared to Newman. Overall, studies with the fluorescence probe suggest that FphE levels are dynamically regulated and are subject to the bacterial growth state and environment in a strain-specific manner [10]. FphE was also shown to be upregulated comparing *S. aureus* exponentially and stationary phases in media in an S9 human bronchial epithelial cell line infection model while being downregulated in a murine pneumonia model [18].

### FphF - SAOUHSC_02962

WhatsGNU analysis on all publicly available *S. aureus* genomes showed an extremely conserved FphF sequence within individual *S. aureus* clonal complexes but varied between each complex (GNU scores of 2218 or 3370 of 10,350). Across bacterial species, putative FphF sequences are highly sequence divergent, while clustering to the expected tree of life [7]. Interestingly, some species like the commensal *S. epidermidis* reference strain RP62A contain two FphF homologs, something only observed for FphF among the Fph proteins [19].

FphF is the only Fph protein for which the atomic structure has been determined. The crystal structure of FphF shows a tetramer, however, in solution FphF forms a dimer. The overall structure represents a typical α/β hydrolase superfamily fold with the core eight β-strands connected by several α helices (Fig. 1C, figure made with UCSF Chimera [22]). The active site is formed by the traditional Ser-His-Asp triad, with apo crystals containing an electron density at the active site interpreted as a sodium ion chelated by two water molecules (PDB ID 6VH9). The active site is exposed to the surface, with one side containing a large hydrophobic pocket and the other side having a polar pocket [6]. A later study also independently determined the apo structure of FphF (PDB ID 7L0A). Magnesium ions were modelled at the active site, similar to the positions of the mentioned sodium. Otherwise, both structures closely resemble each other [7].

Independent studies have characterized FphF as a carboxylesterase. The first study identified, using fluorogenic substrates, that FphF cleaved lipid ester substrates but was unable to process phosphate, phosphonate, or glycosidic substrates. Towards hydrophobic saturated lipid substrates, a promiscuous specificity profile was observed with activity for 4-methylumbelliferyl-acetate to decanoate with the highest activity towards heptanoate [6].

Another study showed FphF activity against 4-nitrophenyl-acetate and butyrate with no activity against 4-nitrophenyl-trimethylacetate, and a lack of glyoxalase activity using the substrate S-lactoylglutathione. In addition, FphF was identified to remove one pivaloyloxymethyl moiety from the carboxy ester prodrug POM-HEX, with a second moiety removed in concert by the hydroxyacylglutathione hydrolase GloB in *S. aureus*, as well as *in vitro*, to activate the prodrug. Prodrug activation was uniform across the population in all observed bacterial cells, demonstrating ubiquitous expression of FphF. Screening of a 32-compound ester substrate library identified that FphF had the highest activity toward oxygen ethers with a preference for unbranched substrates with little regard for chain length or the end-of-chain bulk. Branching at the position following the ester carbonyl was deleterious to FphF activity and when oxygen is included in the chain, positioning at the β-position to the carbonyl is strongly preferred over the γ-position [7].

The substrate preference was further confirmed via an atomic substrate-bound structure (PDB ID 6WCX). Here the heptyl acyl moiety of the preferred model substrate 4-methylumbelliferyl-heptanoate is bound to the active site serine inside the now fully occupied hydrophobic pocket. In conjunction with docking studies, the preference for intermediate sized acyl groups is evident as shorter chains never correctly positioned over the active site and longer chains do not fit [6].

The biological function of FphF is unknown. In various databases, it is currently annotated as an S-formylglutathione hydrolase, which mediates detoxification of cellular formaldehyde, based on studies on the *E. coli* enzyme FrmB [23]. However, the two proteins only share 25 % sequence identity. Major differences are evident when comparing the structure of FphF to other S-formylglutathione hydrolases, like the 24 % sequence homolog to FphF and 60 % sequence homolog to FrmB from *Neisseria meningitides* [24]. The narrow active site environment of S-formylglutathione hydrolases with C2 substrate preference is clearly distinguishing from FphF. The structural observations in combination with the promiscuous esterase activity profile suggest that FphF forms a new esterase subfamily [6].

For FphF a highly specific covalent sulfonyl fluoride-based inhibitor JCP678 was discovered [4] and inhibition was also confirmed *in vitro* [6]. A triazole-urea based inhibitor screen also resulted in specific hits with the regio-isomers KT129 and KT130 (Fig. 1D) [10]. Atomic structures (PDB ID 6VHD KT129-bound and 6VHE KT130-bound) show that the 2-phenylpiperidine-1-carbonyl moiety of these inhibitors binds similar to the hydrophobic lipids. The phenylpiperidine fits well into the hydrophobic pocket of FphF. These inhibitor-bound structures offer a blueprint of compound design targeting FphF [6].

### FphG - SAOUHSC_01912

As mentioned FphG characterizations with biotinylated and fluorescent probes were more difficult compared to the other Fph proteins [4]. It is known that FphG is able to bind β-lactam probes [20, 21], but not much else is known about the protein, except that it is highly conserved within staphylococcal species [19]. Structure prediction indicates that FphG has the greatest diversity of residues predicted to form a narrow acyl pocket which appears to be designed for a specific substrate [6].

### FphH - SAOUHSC_00802

The fphH transposon mutant was the only Fph *S. aureus* single knockout mutant that strongly suggested a functional link to the other Fph proteins, as a gel-based labelling assay suggested upregulation of several other Fph proteins, for example, FphE and FphD [4]. Interestingly, this response to a lack of FphH was not observed in all cells in the population, and the increase of FphE was more significant in the *S. aureus* Newman strain compared to the USA300 strain [10]. These phenomena were observed in the late stationary phase, suggesting a particular relevance of FphH activity during this phase [10]. Apart from the mentioned high conservation of FphH in staphylococcal species, [19] not much else is known about FphH. Structure prediction indicates a lack of a well-defined acyl binding pocket compared to the larger Fph proteins [6].

### FphI - SAOUHSC_00417

FphI might be a distant homolog of FphH, as these two are the only Fph proteins that share more than 25 % sequence identity at 28 %, and they are predicted to fall into the same Pfam family (Fig. 1B). Similar to FphH, not much is known about FphI. It showed moderate upregulation in an S9 human bronchial epithelial cell line infection model and was detected in a murine pneumonia model [18].

### FphJ - SAOUHSC_02824

FphJ with 22 kD is significantly smaller than the other Fph proteins but appears to still form a typical α/β hydrolase domain (Fig. 1B). The active site is predicted to be the least defined of all Fph proteins due to the reduced size of active site helices [6].

## Conclusions

For the development of targeted probes for localization and risk stratification of *Staphylococcus spp.* infections FphB is the most promising newly discovered active serine hydrolase in *S. aureus*. This is due to its presence on the bacterial surface during infections and the preliminary results of several probes and inhibitors for FphB. FphF is the best target for intracellular probes as it appears FphF is expressed at high levels during the bacterial life cycle and can easily be targeted with inhibitors. In addition, library screening hits and inhibitor-bound structures are already available to serve as a blueprint for further development. But FphF also has the potential to be used in prodrug development. FphB and FphE are new therapeutic targets for the treatment or management of Staphylococcus infections. For these and all other Fph proteins, further characterizations are needed to determine their exact biological roles. It is highly likely that phenotypes relevant to the clinics are hidden by redundancy mechanisms within this family of serine hydrolases.
